# Significance of propolis administration for homeostasis of CD4^+^CD25^+^ immunoregulatory T cells controlling hyperglycemia

**DOI:** 10.1186/2193-1801-3-526

**Published:** 2014-09-15

**Authors:** Muhaimin Rifa’i, Nashi Widodo

**Affiliations:** Biology Department, Faculty of Mathematics and Natural Sciences, The University of Brawijaya, Jl Veteran, Malang, 65145 Indonesia

**Keywords:** Diabetes, Mice, Propolis, S961 Peptide, Regulatory T cells

## Abstract

In the present study, we examined the effect of ethanolic soluble derivative of propolis (EEP) extract on immunological function in diabetic mouse models with the aim of highlighting the role of regulatory T cell, the change of cell surface molecule, and in vivo productions of IFN-γ. Murine models of diabetes mellitus (DM) were created by injecting normal mice with S961 peptide. Normal BALB/c mice were injected intraperitoneally with peptide S961 300 mg/kg body weight (BW) twice a day for eight days. On day 15, the spleen was isolated; then, cell surface molecules and regulatory T cells were analyzed using flow cytometry. The histopathological changes were monitored in the liver of treated and control mice. Afterward, we tested the ability of propolis as an immunomodulator that initiate normality metabolism and homeostasis. The propolis decreased blood sugar levels and increased the number of naïve T cells expressing CD62L molecule by suppressing the development of effector cells in diabetic mice. However, the propolis stimulated development of effector cells, which was indicated by increasing the number of CD4^+^CD25^+^ T cells in normal mice. Moreover, the propolis increased the production of IFN-γ in normal mice, whereas in mouse models of diabetes mellitus propolis tends to suppress the production of IFN-γ. The histological analysis of the liver shows that at a dose of 50–200 mg/kg BW propolis does not show a toxic effect so that the dose is categorized safe. Therefore, the ethanolic soluble derivative of propolis (EEP) extract warrant for further exploited as an antidiabetic agent that safe for human.

## Introduction

Diabetes mellitus is the worst and fastest growing metabolic disorder in the world. Now it is known that the heterogeneity of this disorder also increases so that an appropriate therapy becomes a significant challenge. Severe metabolic imbalances and non-physiologic changes in many tissues can occur in diabetes, where oxidative stress plays a critical role in the etiology. Reactive oxygen species (ROS) is involved in diabetes, and it contributes oxidative damage, particularly to nerve, liver, heart, kidney, eyes, large and small blood vessels and immunological system (Oršolić and Bašić 2008; Yue et al. 2003; Obrosova et al. 2003; Loprinzi et al. 2014).

Diabetes mellitus is currently stretching across the entire world. It penetrates population not only in poor and developing countries, but also in developed ones. The prevalence of this disease is known to be increased. In every ten seconds, one person dies from diabetes mellitus. Indonesia is one country having the greatest biological biodiversity in the world. It has natural resources that potentially increase the population welfare as well as provide a wide range of materials to cure various diseases (Vikram and Jena 2010; Syamsudin et al. 2008; Kusumawardani 2011; Castaldo and Capasso 2002). So far, the treatment of various diseases including type-2 diabetes mellitus has a dependency on the synthetic drugs whose safety is still being debated. On the other hand, a lot of herbal medicines claimed to relieve and even to cure type 2 diabetes mellitus. Unfortunately, most of traditional medicine society claimed the various herb treatments have an efficacy without scientific evidence. By considering the case of diabetes mellitus, particularly type-2 diabetes that continues to grow, it is necessary to do adequate research to find a useful drug to ameliorate or even to cure the disease (Murata et al. 2004; Khalil 2006; McLennan et al. 2008; Sforcin 2007; Lotfy et al. 2006; El-Sayed et al. 2009; Sartori et al. 2009).

There has been a growing public opinion arguing that propolis can cure diabetes mellitus. Propolis is complex resinous material collected by honey bees from buds and exudates of certain plant sources neighbouring their hives. The main types of flavonoids contained in propolis are pinocebrin, galangin, chrisin, and caffeic acid phenethyl ester. The use of propolis as an alternative healing therapy for type-2 diabetes mellitus has been claimed to alleviate the disease. Previous study states that propolis improves normal homeostasis by balancing the body’s condition through the enhancement of the immune system (Oršolić and Bašić 2008; Yue et al. 2003; Obrosova et al. 2003; Loprinzi et al. 2014; Ganong 2005; Mahler dan Adler 1999; Cetin et al. 2010; Arora et al. 2009; Bailey 1999; Kang et al. 2010; Sforcin 2007). However, the mechanism action of propolis to modulate the immune system to face type-2 diabetes mellitus cannot be explained. Water extracts of propolis have been studied to prevent the destruction of beta cells by inhibiting the activation of IL-1β and NO synthase activity. Administration with water or ethanolic extract of propolis for seven weeks in mice can decrease glucose, triglyceride, and total cholesterol levels in the blood, thus it is alleged that propolis can control glucose levels and modulate glucose and lipid metabolism. Propolis is also alleged to decline the lipid peroxidation output, and to function as a scavenger for free radicals in rat models of diabetes mellitus. Propolis possesses many functions of biological activities and also has used in folk medicine. Administration of propolis to mouse models of DM suggests homeostasis maintenance, so that further activated cell can be controlled by involving regulatory T cells from both CD4 and CD8 T cells (Oršolić and Bašić 2008; Yue et al. 2003; Obrosova et al. 2003; Sforcin and Bankova 2011; Sawicka et al. 2012; Rifa’i et al. 2004; Lee et al. 2008).

CD4^+^CD25^+^ regulatory T cells (T_reg_) are of central importance for the immune tolerance network and, malfunction of this T cell population can either lead to impaired or increased suppression apparently resulting in an array of distinct diseases. The role of CD4^+^CD25^+^ regulatory T cells in diabetes mellitus is still being debated. These cells are reported to produce IL-10, TGF-β, and IL-4 to stop T cells activation. Some investigators reported that activation and proliferation of T_reg_ correlate with their ability to suppress diabetes, suggesting that existence of T_reg_ is important to maintain homeostasis (Bluestone and Tang 2005; Mahmoud and Al-Ozairi 2013; Afzal et al. 2014). However, another investigator state that T_reg_ was not involved in the development of diabetes mellitus (Afzal et al. 2014). In peripheral blood of DM patient showed a significant increase of IFN-γ, TNF-α, and IL-8. These cytokines contribute to aggravate DM patient and in early stage also cause in the development of insulin resistance (Mahmoud and Al-Ozairi 2013; Aoi et al. 2013). In the other hand propolis is known to contain high-level nutrient factor including vitamins, polyphenols, and amino acids that would be expected to improve insulin sensitivity. Thus, intake of propolis to decline the expression of inflammatory molecules is one of strategies to ameliorate patient with hyperglycemia. The present study was designed to investigate the effect of ethanolic soluble derivative of propolis extract on T cell activation, blood glucose uptake, apoptosis of splenic cell, regulatory T cell development, and IFN-γ production.

## Results and discussion

**Figure 1:**
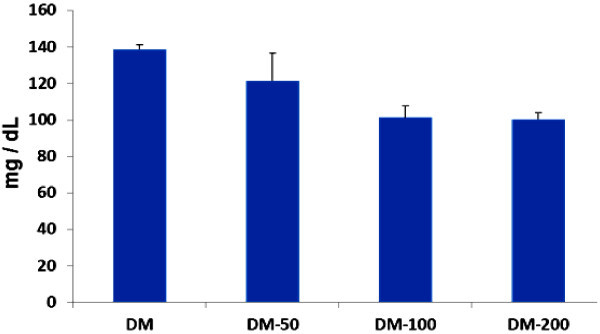
**Propolis could decrease blood sugar levels.** Mouse models of diabetes mellitus (DM) were orally administered by propolis. It was given once a day at doses of 50, 100, or 200 mg/kg BW indicated in the picture. Blood sugar level was measured every other day, and the figure above reported the mean for seven blood measurements.

S961 peptide had an ability to bind with insulin receptor or insulin receptor antagonist. Giving S961 peptide for eight consecutive days might increase blood sugar levels. It was caused by the loss of insulin receptor function blocked by S961. The loss of insulin receptor function would lead to hyperglycemia and also hyper-insulinemia (Vikram and Jena 2010). The increase of blood sugar levels had been seen since two days after the injection of S961 peptide. A high level of blood sugar would be more stable six days after injection of S961 peptide. DM mice treated daily with propolis at doses of 50, 100, or 200 mg/kg BW showed a decrease in blood sugar levels (Figure 1). At doses of 100 to 200 mg/kg BW showed a decrease in blood sugar levels significantly (P < 0.05) compared to controls. The reduction blood sugar levels after treatment with propolis showed that cells were able to metabolize sugar even though insulin receptor was blocked. Although mechanism of action by which propolis can reduce blood glucose level is still illusive, but some investigators suggest that propolis improve glucose uptake by increasing pH of ascites and metabolic tissues (Aoi et al. 2013; McCarty 2005; Cameron et al. 2006; Maalouf et al. 2007; Hayata et al. 2014). In general, diabetes patients have body fluid with acidic condition due to elevation of ketone body production, and improvement of pH can ameliorate healthy condition (Hayata et al. 2014; Cameron et al. 2006; Maalouf et al. 2007; McCarty 2005; Marunaka et al. 2014). Therefore, we speculated that the declined level of blood glucose could be caused by increasing insulin receptor sensitivity due to increasing pH. Moreover according to Aoi et al. ( 2013) dietary propolis elevated buffering capacity in tissue or suppress the production of organic acid so that propolis may improve insulin sensitivity by preventing metabolic acidosis.

**Figure 2:**
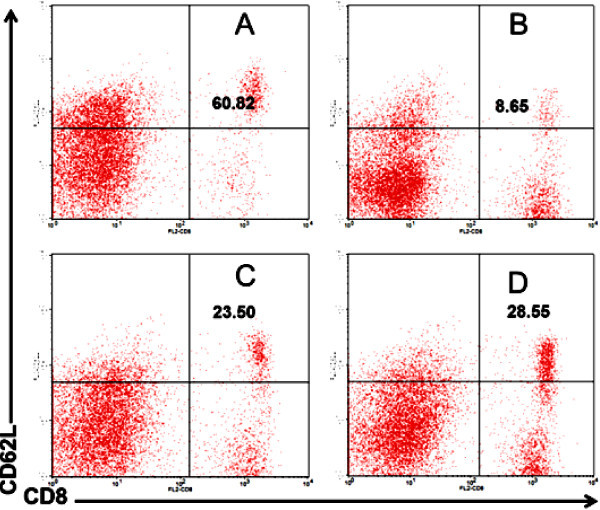
**Propolis in normal mice promote T cell activation but in mouse models of DM it prevented further cell activation. A)** Normal mice as a negative control. **B)** Normal mice treated by propolis at dose of 200 mg/kg BW. **C)** Mouse models of diabetes mellitus (DM) as a positive control. **D)** DM mice treated by propolis at dose of 200 mg/kg BW.

Administration of propolis in normal mice induced T cell activation. Normal mice receiving propolis with a dose of 200 mg/kg BW showed an increase activated T cells, CD8^+^CD62L^-^. Figure 2 showed that naïve T cells in control was 60.82% whereas the number changed into 8.65% after the mice received propolis in dose of 200 mg/kg BW. There were several possibilities that cause naïve T cells shift onto memory types. First, propolis was suspected able to trigger T cell proliferation and activation so that the cell lost CD62L molecules. Second, propolis might stimulate T cells to produce cytokines and such activated T cells lost their CD62L molecule. Interestingly in DM mice, propolis could increase the number of naïve T cells. As shown in Figure 2, naïve T cells changed from 23.50% to 28% after receiving propolis. It was suggested that propolis prevented further activation in order to reach better homeostasis in DM mice. In general, activated T cells will lose CD62L molecules and increase the expression of CD44 and CD69 molecules. CD69 is a cell surface protein synthesized rapidly about two hours after the activation. In diabetic patients, T cell activation can be triggered by the recognition of self-antigen by cytotoxic T and Th1 T cells. T cell activation in turn triggers the activation of other cells involved in the immune system resulting in systemic homeostasis disorders (Rifa’i et al. 2004, 2008; Lee et al. 2008; Shi et al. 2009).

**Figure 3:**
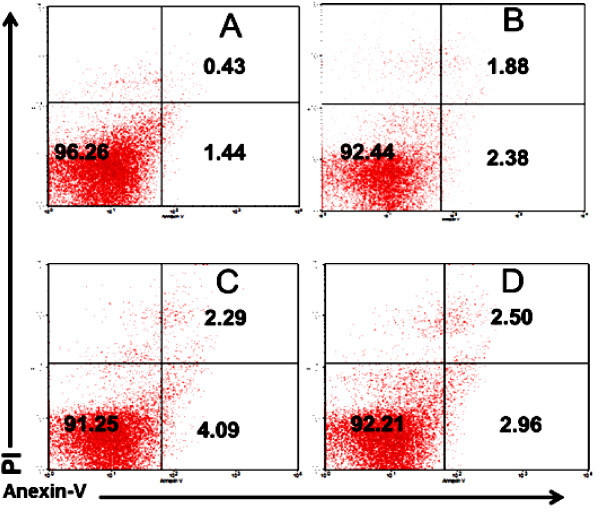
**In normal mice, propolis triggered apoptosis in spleen cells but in mouse models of diabetes mellitus it prevented apoptosis. A)** Normal mice as a negative control. **B)** Normal mice treated by propolis at dose of 200 mg/kg BW. **C)** Mouse models of diabetes mellitus (DM) as a positive control. **D)** DM mice treated by propolis at dose of 200 mg/kg BW.

To know the bioactivity of propolis in cell life cycle we observed the degree of apoptotic cells in the lymphocyte population. The data showed that the number of T cells undergoing apoptosis in the healthy mice treated by propolis was increased. The increasing of apoptosis cells was alleged to offset the high proliferation activity in order kept in balance of the total number of T cells. The opposite phenomenon occurred in DM mouse models. In DM mice, spleen cells had a higher rate of apoptosis compared to healthy mice (Figure 3). It was thus quite plausible that *in vivo* environment of DM mice had a situation to force spleen cells undergoing apoptosis. The apoptosis suggested to prevent further damage caused by activated T cells. Further, analysis showed that administration of propolis in DM mice was known to decrease the rate of apoptosis. This result relates to the evidence that propolis can prevent excessive cell activation. As we have already explained above, after the addition of propolis, T cell surface molecule of lymphocyte is dominated by CD62L (Figure 2) (Rifa’i et al. 2004; Lee et al. 2008).

**Figure 4:**
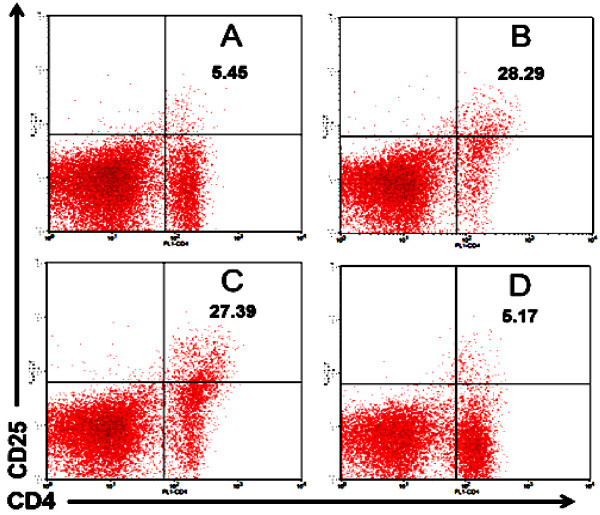
**CD4** ^**+**^ **CD25** ^**+**^ **T cells increased in normal mice that received propolis and vice versa they reduced in mouse models of DM. A)** Normal mice as a negative control. **B)** Normal mice treated by propolis at dose of 200 mg/kg BW. **C)** Mouse models of diabetes mellitus (DM) as a positive control. **D)** DM mice treated by propolis at dose of 200 mg/kg BW.

Regulatory T cells, CD4^+^CD25^+^ T, have a crucial role both in infectious and degenerative diseases. In many cases, CD4^+^CD25^+^ T cells have impotent roles to maintain normal homeostasis. However, the role of regulatory T cells in diabetes mellitus is still unclear. To investigate the role of regulatory T cells in diabetes mellitus in this study we examined the expression of CD25 molecules on CD4 T cells. In the present study, we found that the intake of propolis increase the number of CD4^+^CD25^+^ T cells compared with control, suggesting that dietary propolis promote T cell activation and proliferation (Figure 4). Therefore, we could not explain whether the CD25 molecule expressed on CD4 T cells was the sign of increase in regulatory T cells. According to Rifa’i et al. ( 2004) and Rifa’i ( 2010), the emergence of CD25 molecules can occur if cells are activated and proliferate. CD25 molecule is IL-2Rα which will be synthesized by proliferating cells. Proliferating cells utilize IL-2, so that the presence of IL-2 receptor is required for the cells that are being actively proliferated. Thus, the presence of CD25 molecules does not necessarily indicate an increase in regulatory T cells. However, the emergence of CD25 molecules remains a very interesting phenomenon because in general CD25 molecule can be used as a marker of regulatory T cells even though another marker such as FOXP3 should be included (Shi et al. 2008, 2009; Rifai’i 2010; Lee and Rifa’i 2011).

It is very interesting that the CD4^+^CD25^+^ T cells were increased in the mouse model of diabetes mellitus. There are some possibilities why CD4^+^CD25^+^ T cells increase in the mouse model of diabetes mellitus. First, an increase of CD4^+^CD25^+^ T cells is a manifestation of regulatory T cell enhancement to prevent further cell activation. This fact is consistent with the data shown by Figure 1 in which T cells in mice model of diabetes mellitus were dominated by lymphocytes that lose CD62L molecule, so that the suppressor cells were required to overcome the activated cells. Second, the increase in T cells with CD4^+^CD25^+^ marker may be due to the cells being activated and proliferating so that they require CD25 molecule to bind IL-2. Between the two possibilities above, the second possibility may be closer to the truth. This idea is supported by evidence that administering propolis to mice model of diabetes mellitus decreases the expression of CD25 molecules. This fact is consistent with data in Figure 2 showing that the administration of propolis led to naïve cells (CD62L) were more dominant. The domination of naïve cells reflects that the homeostasis in an individual is running well so that mature T cells migrating from thymus to peripheral lymphoid tissue are not activated. The reduction of activated T cells in DM mice after receiving propolis is corresponding with rules of physiological framework. The activated cells will undergo apoptosis when the cells are no longer needed (Rifai’i 2010; Lee and Rifa’i 2011).

**Figure 5:**
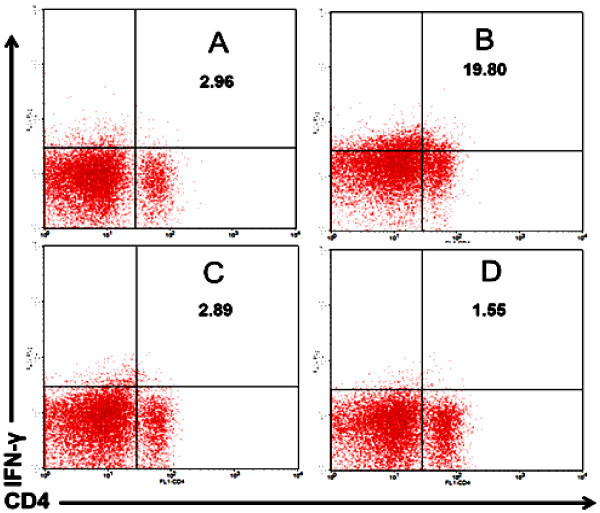
**Administration of propolis in normal mice increased the expression of IFN-γ, but not in mouse models of DM. A)** Normal mice as a negative control. **B)** Normal mice treated by propolis at dose of 200 mg/kg BW. **C)** Mouse models of diabetes mellitus (DM) as a positive control. **D)** DM mice treated by propolis at dose of 200 mg/kg BW.

In normal mice, the administration of propolis increased the expression of interferon gamma (IFN-γ). In contrast, mouse models of DM did not show an increase in IFN-γ production when propolis was administered (Figure 5). The phenomenon why mice model of DM resistant to propolis could not be described in this experiment. There was a possibility that blocking insulin receptor with S961 peptide contribute to this phenomenon (Vikram and Jena 2010). In certain stages IFN-γ was indispensable for the body’s defense system, primarily to cope viral replication and increased the expression of MHC class I and II (Rifa’i et al. 2004). It could thus be expected that the increase of IFN-γ in normal mice after receiving propolis would strengthen their immune system. Slight increase of IFN-γ production in normal mice after administering propolis did not bother normal homeostasis but rather improved the quality of the immune system. However, we presume that propolis acts as a medicinal agent without involving the role of IFN-γ directly (Nakamura et al. 2011).

## Conclusion

Administration of Ethanolic soluble derivative of propolis (EEP) extract to diabetic mice able to decrease of blood sugar levels, suppress the development of effector cells and production of IFN-γ. The propolis also does not show a toxic at a dose of 50–200 mg/kg BW that warrant for an antidiabetic agent that safe for human.

## Materials and methods

### Mice

In this study we used 8-weeks-old BALB /c, which were maintained in the pathogen free facility, Biology Department, Faculty of Sciences, Brawijaya University, Malang, Indonesia.

### Preparation of ethanol extract of propolis

Propolis was obtained from Lawang, East Java, Indonesia. GC-MS analysis has shown that Ethanolic propolis extract (EEP) contained (percentage of total ion current): Benzoic acid 0.41, Phenylic acid 95.62, D-glucofuranuronic acid 0.56, 4-oxo-2-thioxo-3-thiozolidinepropionic acid 0.79, 1-Naphtalenemethanol 95.62, Patchoulene 0.27, D-mannitol 0.51, Threitol 0.86, Glycerol 0.86 (Syamsudin et al. 2009). Preparations of propolis extract consisted of three phases including drying, extracting, and evaporating. The drying process began by washing the sample, cutting it into small pieces, and putting them in the oven with a temperature of 40–60°C. Before the extraction process, samples were dried and then crushed by a blender. 100 grams of dry samples were weighed and put in 1 L Erlenmeyer glass, soaked with ethanol to the volume of 1 L. Sample in ethanol was stirred for ± 30 minutes and allowed to stand overnight to settle. Then, solution containing the active substance was filtered with filter paper. Soaking process was repeated three times and the last stage was evaporation. Extraction solvent was inserted into 1 L evaporation flask. Then, water bath was filled with water up to a full circuit and then installed according to an equipment protocol and set to a temperature of 90°C. Ethanol was allowed to drip in the flask (±1.5–2 hours/flask containing ± 900 mL). Extraction results obtained roughly one tenth of dried natural materials (ten grams extract/100 gram’s sample).

### Induction of type 2 diabetes mellitus with peptide S961 and propolis treatment

Normal BALB/c mice were injected intraperitoneally with peptide S961 300 mg/kg BW twice a day for eight days. Mice were divided into five groups including positive and negative control groups. The dose of propolis each 50, 100, and 200 mg/kg respectively was administered by oral gavage to BALB/c mice once a day from the first day of peptide injection until the day 14. Glucose level was measured every other day. On day 15, the spleen was isolated, then cell surface molecules, intracellular cytokine, and regulatory T cells were analyzed by flow cytometry.

### Oral treatment with dextrose and sucrose

Mouse models of diabetes mellitus received dextrose (2 g/kg BW) by force-fed to maximize diabetes condition. 150 grams of pure sugar/sucrose were put into the Erlenmeyer flask added with 1350 mL of distilled water and shaken to homogenize the solution. Sucrose solution was given to groups of mouse models of diabetes mellitus as they had *ad libitum* drinking. Sucrose solution was given simultaneously with the injection of peptide and replacement of sucrose solution was performed daily for two weeks.

### Measurement of blood sugar levels

Measurement of blood sugar level was done every other day with *One Touch Glucometer*. Mice were put into a trap, and the blood was taken from their tails. Blood was dropped into a glucostick screen installed on glucometer and noted within 5 seconds.

### Isolation of lymphoid cells and flow cytometry analysis

Spleen was washed with sterile PBS twice and put on petri dish containing sterile PBS. Spleen organ was pressed by using syringe holder. Single cell solution was filtered with a sterile wire and put into a 15 mL polypropylene. Suspension in polypropylene was added with PBS up to 10 mL and then put in a centrifuge (2500 rpm, 4°C for five minutes). Then the supernatant was discarded, and the obtained pellet was resuspended with 1 mL of sterile PBS. Single cell suspension containing 2 ~ 3 × 10^6^ cells was washed with PBS and stained with FITC-conjugated anti-mouse CD4, PE-conjugated anti-mouse CD8, PE-conjugated anti-mouse CD25, PE-conjugated anti-mouse CD62L and anti- mouse CD62L (Rifa’i et al. 2004; Bluestone and Tang 2005).

### Intracellular staining

Intracellular cytokine staining was performed with a Cytofix/Cytoperm kit (BD-Biosciences Pharmingen) according to the protocol provided by the manufacturer. Cells were incubated with FITC-conjugated anti-mouse CD4, PE-conjugated anti-mouse CD8, PE-Cy5-conjugated anti-mouse anti-interferon (IFN)-γ antibodies. Pellets with approximately 2 ~ 3 × 10^6^ cells were stained with FITC-conjugated anti-mouse CD4, PE-conjugated anti-mouse CD8 for 30 minutes. After incubation, the suspension was washed, and pellet was resuspended in cytofix buffer (200 μL) for 20 minutes in dark conditions, 4°C, then resuspended in 1 mL wash-perm and centrifuged again at 2500 rpm at 4°C for 5 minutes. Supernatant was discarded, and the obtained pellet was subjected to intracellular staining with anti-mouse anti-interferon (IFN)-γ for 30 minutes.

### Examination of apoptotic cells

Double staining for cellular DNA using propidium iodide (PI) and FITC- conjugated annexin V binding was performed as follows. After washing twice with PBS, 2 ~ 3 × 10^6^ cells were resuspended in annexin-V binding buffer and FITC-conjugated annexin-V was added to a final concentration of 1 μg/mL cell suspension. PI (10 μg/mL in annexin-V binding buffer) was added 15 minutes before running in Flow Cytometry. PI concentration was adjusted in a final concentration of 1 μg PI/mL cell suspension. The mixture was incubated for 15 minutes in the dark at room temperature and then analyzed with Flow Cytometry (Caliber using CellQuest program).

### Statistical analysis

The obtained data were analyzed by CellQuest software and tested by statistical analysis ANOVA (Analysis of Variance) with P < 0.05. All results were presented as mean of ± SD values of five mice in each group.

## References

[CR1] Afzal N, Javaid K, Zaman S, Zafar A, Abdul H, Nagi AH (2014). Enumeration of CD4^+^CD25^+^T regulatory cells in type-II diabetes retinopathy. Pak J Pharm Sci.

[CR2] Aoi W, Hosogi S, Niisato N, Yokoyama N, Hayata H, Miyazaki H, Kusuzaki K, Fukuda T, Fukui M, Nakamura N, Marunaka Y (2013). Improvement of insulin resistance, blood pressure and interstitial pH in early developmental stage of insulin resistance in OLETF rats by intake of propolis extracts. Biochem Biophys Res Commun.

[CR3] Arora S, Ojha SK, Vohora D (2009). Characterization of Streptozotocin Induced Diabetes Mellitus In Swiss Albino Mice. Global J Pharmacol.

[CR4] Bailey CJ (1999). Insulin resistance and antidiabetic drugs. Biochem Pharmacol.

[CR5] Bluestone JA, Tang Q (2005). How do CD4^+^CD25^+^ regulatory T cells control autoimmunity?. Curr Opin Immunol.

[CR6] Cameron MA, Maalouf NM, Adams-Huet B, Moe OW, Sakhaee K (2006). Urine composition in type 2 diabetes: predisposition to uric acid nephrolithiasis. J Am Soc Nephrol.

[CR7] Castaldo S, Capasso F (2002). Propolis, an old remedy used in modern medicine. Fitoterapia.

[CR8] Cetin E, Silici S, Cetin N, Guclu BK (2010). Effects Of Diets Containing Different Concentration of Propolis On Hematological And Immunological Variables In Laying Hens. Poult Sci.

[CR9] El-Sayed e-SM, Abo-Salem OM, Aly HA, Mansour AM (2009). Potential Antidiabetic And Hypolipidemic Effects Of Propolis Extract In Streptozotocin-Induced Diabetic Rats. Pak J Pharm Sci.

[CR10] Ganong FG (2005). Endocrine functions of the pancreas & regulation of carbohydrate metabolism. Review of Medical Physiology.

[CR11] Hayata H, Miyazaki H, Niisato N, Yokoyama N, Marunaka Y (2014). Lowered extracellular pH is involved in the pathogenesis of skeletal muscle insulin resistance. Biochem Biophys Res Commun.

[CR12] Kang L-J, Lee HB, Bae H-J, Lee S-G (2010). Antidiabetic effect of propolis: reduction of expression of glucose-6-phosphatase through inhibition of Y279 and Y216 autophosphorylation of GSK-3α/β in HepG2 cells. Phytother Res.

[CR13] Khalil ML (2006). Biological activity of bee propolis in health and disease. Asian Pac J Cancer Prev.

[CR14] Kusumawardani RK (2011). Detection of IGF-1 in serum of type-2 diabetes mellitus patient. Biology Dept, Faculty of Sciences, Brawijaya University.

[CR15] Lee YH, Rifa’I M (2011). CD4^+^CD25^+^ FOXP3^+^ Regulatory T Cells In Allogeneic Hematopoietic Cell Transplantation. Journal of Tropical Life Science.

[CR16] Lee YH, Ishida Y, Rifa’i M, Shi Z, Isobe K, Suzuki H (2008). Essential role of CD8^+^CD122^+^ regulatory T cells in the recovery from experimental autoimmune encephalomyelitis. J Immunol.

[CR17] Loprinzi PD, Hager KK, Ramulu PY (2014). Physical activity, glycemic control, and diabetic peripheral neuropathy: A national sample. J Diabetes Complicat.

[CR18] Lotfy M, Badra G, Burham W, Alenzi FQ (2006). Combined use of honey, bee propolis and myrrh in healing a deep, infected wound in a patient with diabetes mellitus. Br J Biomed Sci.

[CR19] Maalouf NM, Cameron MA, Moe OW, Adams-Huet B, Sakhaee K (2007). Low urine pH: a novel feature of the metabolic syndrome. Clin J Am Soc Nephrol.

[CR20] Mahler RJ, Adler ML (1999). Type 2 Diabetes Mellitus: Update On Diagnosis, Pathophysiology, And Treatment. Clinical Review 102. J Clin Endocrinol Metab.

[CR21] Mahmoud F, Al-Ozairi E (2013). Inflammatory Cytokines and the Risk of Cardiovascular Complications in Type 2 Diabetes. Dis Markers.

[CR22] Marunaka Y, Yoshimoto K, Aoi W, Hosogi S, Ikegaya H (2014). Low pH of interstitial fluid around hippocampus of the brain in diabetic OLETF rats. Mol Cell Ther.

[CR23] McCarty MF (2005). Acid–base balance may influence risk for insulin resistance syndrome by modulating cortisol output. Med Hypotheses.

[CR24] McLennan SV, Bonner J, Milne S, Lo L, Charlton A, Kurup S, Jia J, Yue DK, Twigg SM (2008). The anti-inflammatory agent Propolis improves wound healing in a rodent model of experimental diabetes. Wound Repair Regen.

[CR25] Murata K, Yatsunami K, Fukuda E, Onodera S, Mizukami O, Hoshino G, Kamei T (2004). Antihyperglycemic effects of propolis mixed with mulberry leaf extract on patients with type 2 diabetes. Altern Ther Health Med.

[CR26] Nakamura K, Kawasaki E, Imagawa A, Awata T, Ikegami H, Uchigata Y, Kobayashi T, Shimada A, Nakanishi K, Makino H, Maruyama T, Hanafusa T (2011). Type 1 Diabetes and Interferon Therapy. Diabetes Care.

[CR27] Obrosova IG, Minchenko AG, Vasupuram R, White L, Abatan OI, Kumagai AK, Frank RN, Stevens MJ (2003). Aldose reductase inhibitor fidarestat prevents retinal oxidative stress and vascular endothelial growth factor overexpression in streptozotocin-diabetic rats. Diabetes.

[CR28] Oršolić N, Bašić I, Govil JN, Singh VK (2008). Honey Bee Products and their Polyphenolic Compounds in Treatment of Diabetes. Phytopharmacology and Therapeutic Values IV.

[CR29] Rifa’i M, Kawamoto Y, Nakashima I, Suzuki H (2004). Essential roles of CD8^+^CD122^+^ regulatory T cells in the maintenance of T cell homeostasis. J Exp Med.

[CR30] Rifa’i M, Shi Z, Zhang SY, Lee YH, Shiku H, Isobe K, Suzuki H (2008). CD8^+^CD12^+^ regulatory T cells recognize activated T cells via conventional MHC class I–αβTCR interaction and become IL-10-producing active regulatory cells. Int Immunol.

[CR31] Rifai’i M (2010). Andrographolide ameliorate rheumatoid arthritis by promoting the development of regulatory T cells. Journal of Tropical Life Science.

[CR32] Sartori DRS, Kawakami CL, Orsatti CL, Sforcin JM (2009). Propolis Effect On Streptozotocin-Induced Diabetic Rats. J Venom Anim Toxin incl Trop Dis.

[CR33] Sawicka D, Car H, Borawska MH, Niklinski J (2012). The anticancer activity of propolis. Folia Histochem Cytobiol.

[CR34] Sforcin JM (2007). Propolis and Immune System: A Review. J Ethnopharmacol.

[CR35] Sforcin JM, Bankova V (2011). Propolis: is there a potential for the development of new drugs?. J Ethnopharmacol.

[CR36] Shi Z, Rifa’i M, Lee YH, Shiku H, Isobe K, Suzuki H (2008). Importance of CD80/CD86–CD28 interactions in the recognition of target cells by CD8^+^CD122^+^ regulatory T cells. Immunology.

[CR37] Shi Z, Okuno Y, Rifa’i M, Endharti AT, Akane K, Isobe K, Suzuki H (2009). Human CD8^+^CXCR3^+^ T cells have the same function as murine CD8^+^CD122^+^ Treg. Eur J Immunol.

[CR38] Syamsudin, Dewi RM, Kusmardi (2008). Immunomodulatory and in vivo Antiplasmodial Activities of Propolis Extract. Global J Pharmacol.

[CR39] Syamsudin, Wiryowidagdo S, Simanjuntak P, Heffen WL (2009). Chemical Composition of Propolis from Different Regions in Java and their Cytotoxic Activity. Am J Biochem Biotech.

[CR40] Vikram A, Jena G (2010). S961, an insulin receptor antagonist causes hyperinsulinemia, insulin-resistance and depletion of energy stores in rats. Biochem Biophys Res Commun.

[CR41] Yue KK, Chung WS, Leung AW, Cheng CH (2003). Redox changes precede the occurrence of oxidative stress in eyes and aorta, but not in kidneys of diabetic rats. Life Sci.

